# Draft Genome Sequences of Two Sporothrix schenckii Clinical Isolates Associated with Human Sporotrichosis in Colombia

**DOI:** 10.1128/genomeA.00495-18

**Published:** 2018-06-14

**Authors:** Oscar M. Gomez, Laura C. Alvarez, Jose F. Muñoz, Elizabeth Misas, Juan E. Gallo, Maria del P. Jimenez, Myrtha Arango, Juan G. McEwen, Orville Hernandez, Oliver K. Clay

**Affiliations:** a Cellular and Molecular Biology Unit, Corporación para Investigaciones Biológicas, Medellín, Colombia; b Institute of Biology, Universidad de Antioquia, Medellín, Colombia; c School of Medicine, Universidad de Antioquia, Medellín, Colombia; d Broad Institute of MIT and Harvard, Cambridge, Massachusetts, USA; e GenomaCES, Universidad CES, Medellín, Colombia; f MICROBA Research Group, School of Microbiology, Universidad de Antioquia, Medellín, Colombia; g School of Medicine and Health Sciences, Universidad del Rosario, Bogotá, Colombia

## Abstract

Sporothrix schenckii is a thermodimorphic fungal pathogen with a high genetic diversity. In this work, we present the assembly and similarity analysis of the whole-genome sequences of two clinical isolates from Colombia of S. schenckii *sensu stricto*.

## GENOME ANNOUNCEMENT

Sporothrix schenckii is a thermodimorphic fungal pathogen and is the etiologic agent of sporotrichosis, a subcutaneous mycosis that affects mammals. The genus Sporothrix includes at least four human-pathogenic species: S. schenckii *sensu stricto*, S. brasiliensis, S. globosa, and S. luriei (1–3). S. schenckii sensu stricto presents a worldwide distribution and high genetic diversity, and five lineages (A to E) have been described for this species to date (4). In South America, the areas where the pathogen is most endemic include Peru, Colombia, Venezuela, and Brazil (4, 5). The primary mode of infection is traumatic inoculation with contaminated decaying plant material (4). Sporotrichosis has a wide spectrum of clinical manifestations, ranging from cutaneous forms to systemic presentations (6). The diagnosis is based on fungal growth and microscopic observation; however, these methods are time consuming, have low sensitivity, and do not allow species differentiation. Nevertheless, continued efforts have improved sensitivity and specificity for diagnostic molecular tests (7). The genus Sporothrix is exceptional in the fungal kingdom due to its high occurrence of outbreaks in areas where the pathogen is endemic and the characteristic differences in those outbreaks (4). Given the wide genetic diversity of the S. schenckii complex and the rapid emergence of sporotrichosis in different countries, the study of the genotypes involved in the different outbreaks is crucial for developing public health strategies to control the disease (5). Despite the efforts of the community studying this mycosis, genomic data reflecting the pathogen’s diversity remain limited. Thus, we obtained isolates from two clinical cases of cutaneous sporotrichosis in human Colombian patients and sequenced the complete genomes.

Genomic DNA for sequencing was prepared from yeast culture, using phenol-chloroform extraction. Library preparation and 150-bp paired-end sequencing were performed using the Illumina HiSeq 2000 platform, generating ∼14 million paired-end reads per strain. The reads were assembled using SOAPdenovo2 version 2.04 and GapCloser for SsEM7 and SPAdes version 3.10 for SsMS1. The assembled scaffolds generated by the two strains were aligned and oriented with MAUVE software. The genomes of the assemblies were processed using the QUAST version 4.5 program, and assembly statistics are shown in Table 1.

**TABLE 1:** Summary of assembly statistics

Sample ID	Genome size (Mb)	Coverage (×)	No. of scaffolds	Scaffold *N*_50_ (bp)	Largest scaffold size (Mb)	Scaffold *L*_50_ (no.)	GC content (%)	GenBank accession no.
SsMS1	32.7	127	124	625,938	1.78	18	54.89	PGUU00000000
SsEM7	32.9	128	181	1,079,862	3.72	9	54.81	NTMI00000000

To identify the genotype of the S. schenckii isolates, we first identified ITS, CAL, TEF1, and TEF3 sequences in the assembly using BLAST searches. This was followed by maximum likelihood based phylogenetic reconstruction using IQtree version 1.4.4 software, which included sequences of these markers from DDBJ/EMBL/GenBank as described by Zhang et al. in 2015. FigTree was used for tree visualization. The two isolates were classified as S. schenckii sensu stricto lineage A, a subgroup consisting of isolates of hyperendemic zones of South America, while the two genomes available for this species reported by Cuomo et al. in 2014 (8) and Teixeira et al. in 2015 (9) are from lineage E, a subgroup that represents isolates from USA. In this work, we report the first two genomes of lineage A of S. schenckii.

### Accession number(s).

The whole-genome sequences of both strains were deposited at DDBJ/ENA/GenBank under the accession numbers cited in Table 1.
